# Synthesis, structure and computational study of di­acetato­bis­(2-amino-6-fluoro-1,3-benzo­thia­zole-κ*N*^3^)zinc(II)

**DOI:** 10.1107/S2056989026009163

**Published:** 2026-09-03

**Authors:** Dilshoda Amanova, Zubaydabegim Radjabova, Sardor Murodov, Sevinch Erkinboyeva, Odil Choriev, Jamshid Ashurov, Shahlo Daminova

**Affiliations:** a Termez Branch of Tashkent State Medical University, 64 Islom Karimov Street, Termiz 132000, Uzbekistan; b National University of Uzbekistan named after Mirzo Ulugbek University Street 4 Tashkent 100174 Uzbekistan; c Uzbekistan-Japan Innovation Centre of Youth, University Street 2B, Tashkent 100095, Uzbekistan; d Institute of Bioorganic Chemistry, Academy of Sciences of Uzbekistan, Mirzo, Ulugbek Street 83, Tashkent 100125, Uzbekistan; Tokyo University of Science, Japan

**Keywords:** crystal structure, 2-amino-6-fluoro-1,3-benzo­thia­zole, Hirshfeld surface, Void analysis, π–π stacking

## Abstract

In the title zinc(II) complex, [Zn(C_2_H_3_O_2_)_2_(C_7_H_5_FN_2_S)_2_], the Zn^II^ atom adopts a tetra­hedral coordination geometry (τ_4_ = 0.96), defined by two nitro­gen atoms from two neutral 2-amino-6-fluoro-1,3-benzo­thia­zole ligands and two oxygen atoms from two monodentately coordinated acetate anions. The crystal packing is governed by classical N—H⋯O hydrogen bonds, which generate one-dimensional chains extending along the [011] direction. These chains are further reinforced by bifurcated N—H⋯(O,F) hydrogen bonds together with C—H⋯π, O⋯π, and offset π–π inter­actions, resulting in a robust three-dimensional supra­molecular framework. Hirshfeld surface analysis reveals that H⋯H (31.5%), C⋯H/H⋯C (16.3%), O⋯H/H⋯O (13.0%), F⋯H/H⋯F (12.9%), and S⋯H/H⋯S (10.2%) contacts make the largest contributions to the crystal packing, while a void analysis confirms the efficient packing of the mol­ecules within the crystal.

## Abstract 2

The title tetra­hedral Zn^II^ complex with 2-amino-6-fluoro-1,3-benzo­thia­zole has been structurally characterized. The crystal packing is consolidated by N—H⋯O, bifurcated N—H⋯(O,F), C—H⋯π, O⋯π and offset π–π inter­actions.

## 1. Chemical context

Benzo­thia­zole derivatives constitute an important class of nitro­gen- and sulfur-containing heterocyclic compounds owing to their structural diversity, coordination versatility and broad spectrum of biological and physicochemical properties (Keri *et al*., 2015 ▸). The presence of nitro­gen and sulfur donor atoms within the fused aromatic framework provides favourable coordination sites for transition-metal ions, while substitution on the benzene ring enables systematic modulation of the electronic properties, mol­ecular polarity and inter­molecular inter­actions of the ligand. Consequently, substituted benzo­thia­zoles have attracted considerable attention in coordination chemistry, crystal engineering and medicinal chemistry (Barbarossa *et al.*, 2023 ▸).

Particular inter­est has been devoted to 2-amino­benzo­thia­zole derivatives because the heterocyclic nitro­gen atom readily coordinates to metal centres, whereas the exocyclic amino group usually remains non-coordinated and serves as an efficient hydrogen-bond donor. This dual functionality facilitates the formation of coordination compounds together with extended supra­molecular architectures sustained by classical and non-classical inter­molecular inter­actions. Previous structural investigations have demonstrated that Zn^II^ complexes containing 2-amino­benzo­thia­zole predominantly exhibit tetra­hedral coordination environments in which the ligand binds through the ring nitro­gen atom rather than the amino N atom (Davarski *et al.*, 1996 ▸; Kim & Kang, 2012 ▸; Suh *et al.*, 2009 ▸).

Fluorinated benzo­thia­zole derivatives are considered particularly promising because fluorine substitution modifies the electronic distribution of the aromatic system without introducing significant steric effects. The strong electron-withdrawing character of fluorine influences the donor ability of the coordinating nitro­gen atom and frequently contributes to crystal stabilization through weak inter­molecular contacts, including N—H⋯F and C—H⋯F inter­actions. In addition, fluorination affects the π-electron density of the aromatic framework, thereby influencing aromatic stacking and other non-covalent inter­actions responsible for crystal packing (Li *et al.*, 2013 ▸; Kumar *et al.*, 2017 ▸).

The mol­ecular structure of free 2-amino-6-fluoro-1,3-benzo­thia­zole has previously shown that fluorine substitution preserves the near-planarity of the benzo­thia­zole skeleton while allowing the formation of an extensive hydrogen-bonding network in the crystal. Such structural characteristics indicate that this ligand is well suited for the construction of coordination compounds in which both coordination and supra­molecular inter­actions contribute to the overall crystal architecture (Jai-nhuknan *et al.*, 1997*a* ▸,*b* ▸).

Mixed-ligand Zn^II^ complexes containing nitro­gen-donor heterocycles and carboxyl­ate ligands have been widely investigated because of their structural diversity and their ability to generate robust supra­molecular assemblies through hydrogen bonding and aromatic inter­actions. In many cases, acetate anions coordinate in a monodentate manner, leaving the second oxygen atom available for inter­molecular hydrogen bonding, which plays an essential role in directing crystal packing (Guo *et al.*, 2011 ▸). Moreover, benzo­thia­zole-based Zn^II^ complexes have attracted increasing attention owing to their potential biological activity and favourable structural characteristics arising from the combination of rigid aromatic ligands and flexible coordination environments (Babu *et al.*, 2015 ▸; Reddy *et al.*, 2014 ▸).
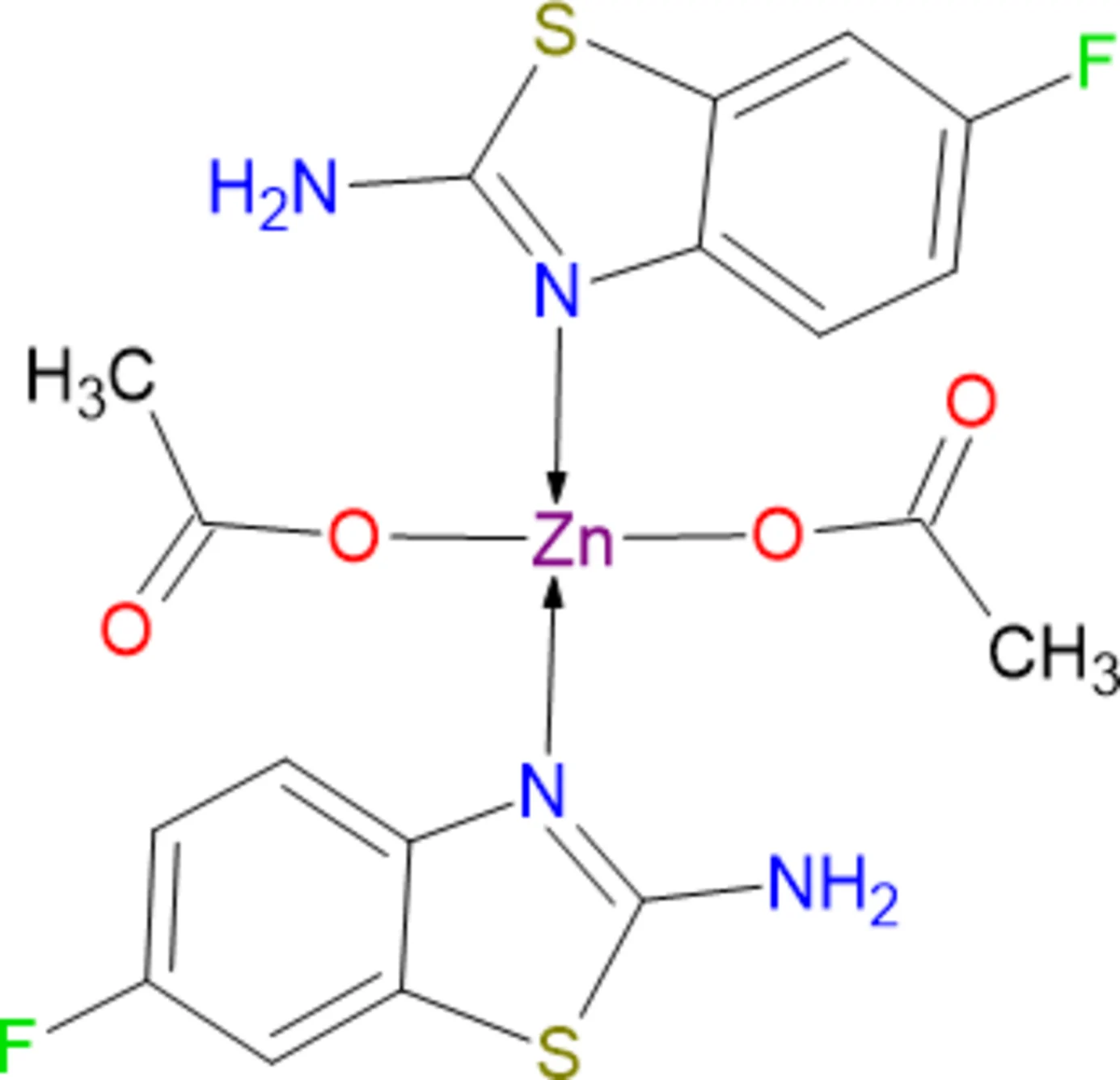


Motivated by these considerations, 2-amino-6-fluoro-1,3-benzo­thia­zole was selected as a ligand combining a rigid aromatic framework, a strong nitro­gen donor atom, an amino group capable of forming inter­molecular hydrogen bonds and a fluorine substituent expected to influence crystal packing. Zinc acetate was employed as the metal precursor because acetate ions readily adopt monodentate coordination while simultaneously providing additional hydrogen-bond acceptor sites. As part of our continuing studies on coordination compounds of substituted benzo­thia­zoles, the title compound, [Zn(C_2_H_3_O_2_)_2_(C_7_H_5_FN_2_S)_2_], (I), was synthesized and its crystal structure, supra­molecular organization, Hirshfeld surface and crystal void distribution were investigated.

## 2. Structural commentary

**Figure 1:**
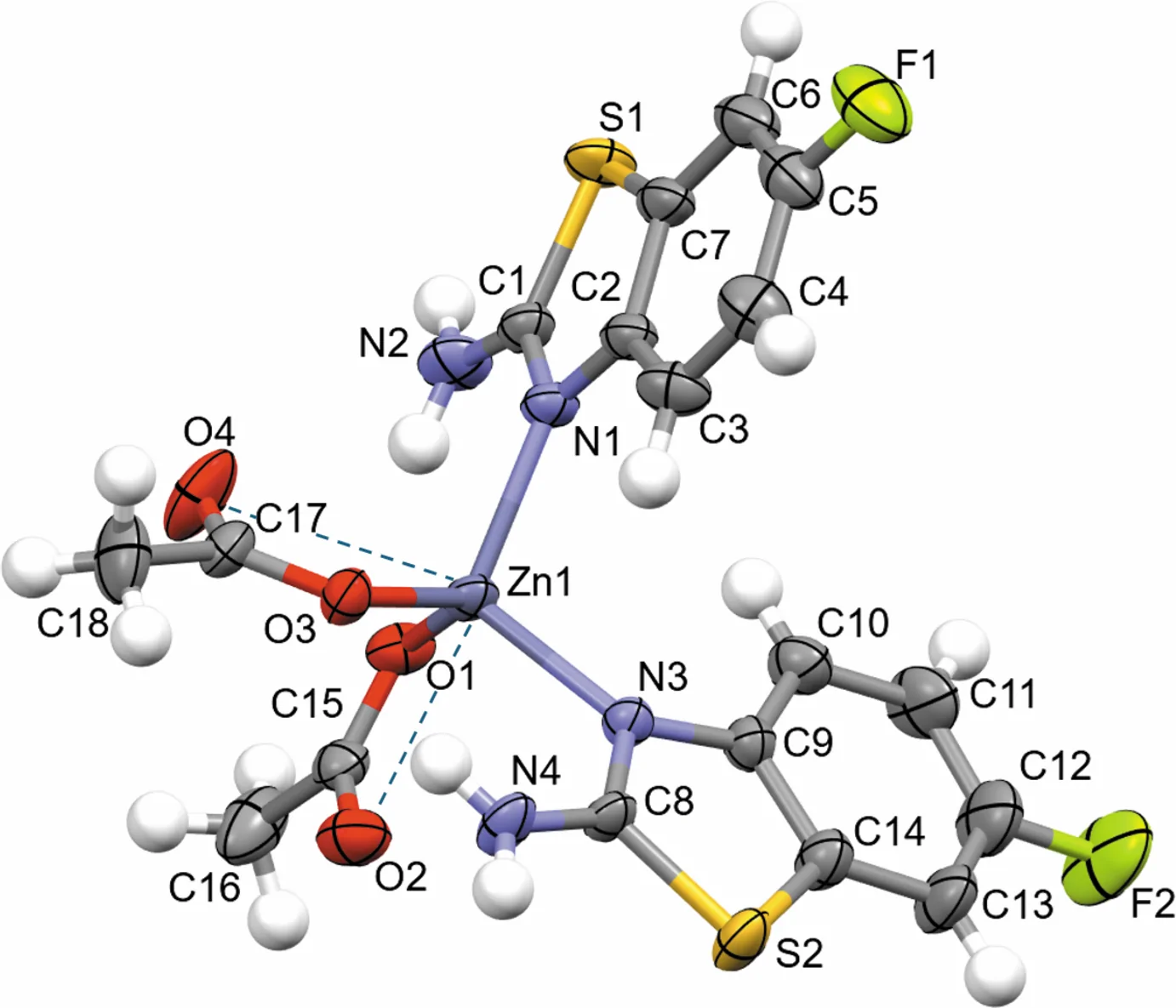
Mol­ecular structure of the title compound, showing the atom-numbering scheme. Displacement ellipsoids are drawn at the 40% probability level.

The asymmetric unit of (I) comprises one independent neutral complex mol­ecule (Fig. 1 ▸). The title compound crystallizes in the non-centrosymmetric Sohncke space group *Pna*2_1_. The refined Flack parameter of 0.002 (12) confirms that the absolute structure has been reliably established. The Zn^II^ centre is coordinated by two nitro­gen atoms from two 2-amino-6-fluoro-1,3-benzo­thia­zole (AFBT) ligands and two oxygen atoms from two acetate (ac) anions, giving a four-coordinate ZnN_2_O_2_ environment.

The AFBT ligands coordinate exclusively through the endocyclic nitro­gen atoms of the thia­zole rings, whereas both ac anions adopt a monodentate coordination mode. The Zn—O and Zn—N bond lengths (Table 1 ▸) are consistent with those reported for structurally related Zn^II^ complexes containing 2-amino­benzo­thia­zole derivatives, in which the Zn—N and Zn—O bond distances generally fall within the ranges 2.02–2.05 and 1.94–1.98 Å, respectively (Tojiboyeva *et al.*, 2025 ▸; Kim & Kang, 2012 ▸; Kim *et al.*, 2009 ▸).

The coordination geometry around the Zn^II^ centre is best described as a slightly distorted tetra­hedron. Qu­anti­tative evaluation using the four-coordinate geometry index τ_4_ proposed by Yang *et al.*, 2007 ▸, defined as τ_4_ = [360 − (α + β)]/141, where α and β are the two largest coordination angles, gives a value of 0.96, indicating a tetra­hedral geometry. The slight distortion from the ideal tetra­hedron arises from the different donor characteristics of the nitro­gen and oxygen atoms together with the spatial arrangement of the AFBT and ac ligands.

Although the *ac* anions coordinate in a monodentate fashion, short secondary contacts of Zn1⋯O2 = 2.674 (3) Å and Zn1⋯O4 = 2.819 (4) Å are observed. These separations are significantly longer than normal Zn—O coordination bonds and therefore cannot be regarded as additional coordination inter­actions. Nevertheless, they indicate the presence of weak secondary intra­molecular Zn⋯O contacts, reflecting the spatial organization of the coordination environment.

Both AFBT ligands remain essentially planar after coordination. The root-mean-square (r.m.s.) deviations from the least-squares planes are 0.019 and 0.020 Å, confirming that the π-conjugated benzo­thia­zole framework is well preserved upon coordination. Furthermore, the geometry of the coordinated ligands closely resembles that of the free AFBT mol­ecule, indicating that coordination to the Zn^II^ centre has little influence on the mol­ecular structure of the heterocyclic fragment (Jai-nhuknan *et al.*, 1997*a* ▸,*b* ▸).

The ac groups exhibit the characteristic asymmetry of the C—O bond lengths expected for monodentate carboxyl­ate ligands. Coordination occurs through only one oxygen atom of each ac anion, while the second oxygen atom remains uncoordinated, resulting in the observed differentiation of the C—O bond lengths.

## 3. Supra­molecular features

**Figure 2:**
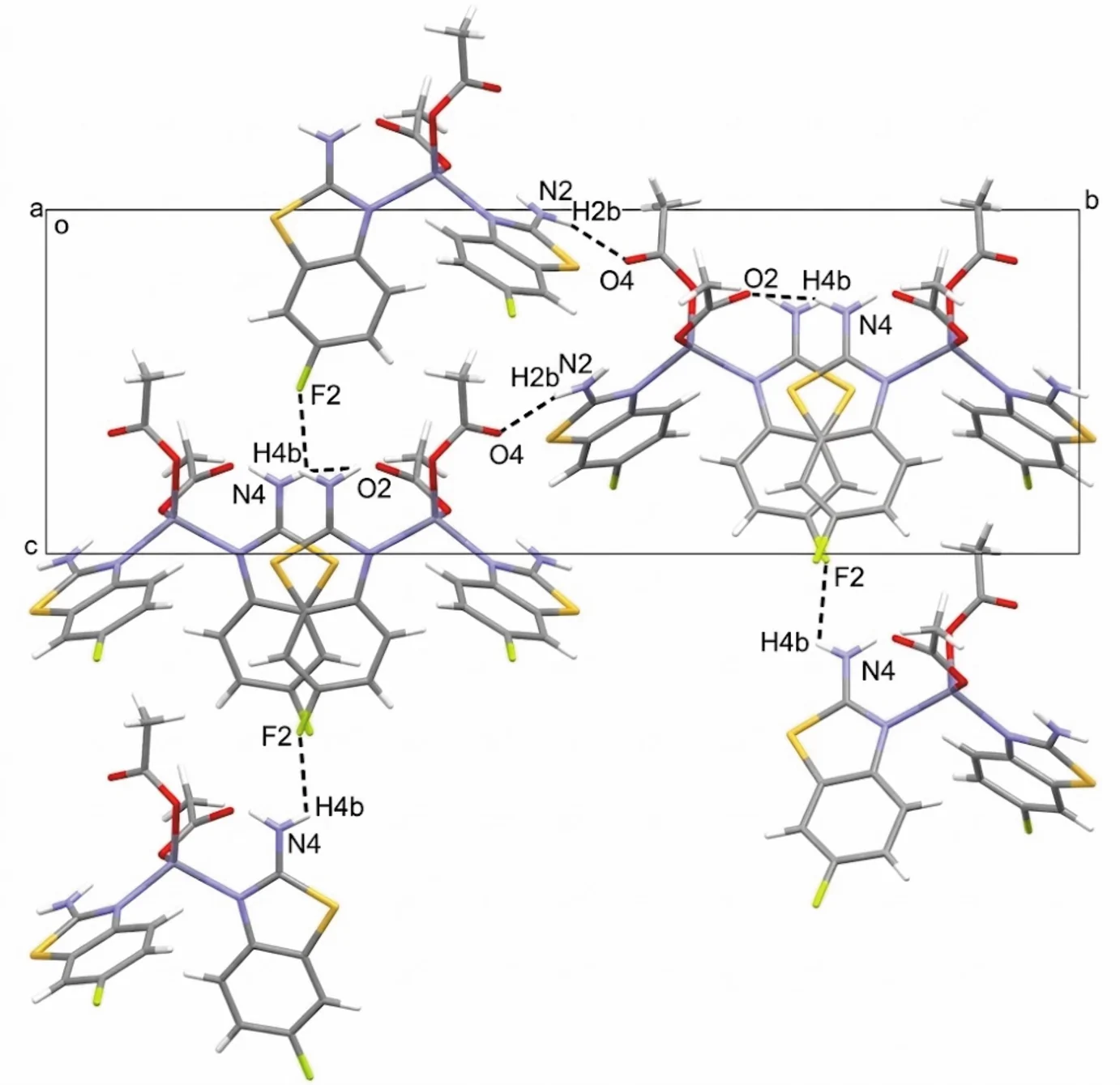
Crystal packing of the title compound viewed along the [011] direction, showing the network of N—H⋯O and N—H⋯F hydrogen bonds. Hydrogen bonds are shown as dashed lines.

The crystal packing is governed by a combination of classical hydrogen bonds and weaker non-covalent inter­actions, which collectively generate a robust three-dimensional supra­molecular architecture (Fig. 2 ▸). The principal contribution to the crystal packing arises from classical N—H⋯O hydrogen bonds formed between the amino groups of the AFBT ligands and the non-coordinating oxygen atoms of the ac anions (Table 2 ▸). In particular, the N2—H2*B*⋯O4 and N4—H4*B*⋯O2 hydrogen bonds link adjacent mol­ecules into infinite chains propagating along the [011] direction.

Besides forming classical hydrogen bonds, atom N4 also participates in a bifurcated hydrogen bond, where hydrogen atom H4*B* simultaneously inter­acts with atoms O2 and F2. The N4—H4*B*⋯F2 contact is considerably weaker than the corresponding N—H⋯O hydrogen bond; however, it plays an important structure-directing role. Although fluorine is generally regarded as a weak hydrogen-bond acceptor, the N—H⋯F inter­action links adjacent hydrogen-bonded chains and reinforces the overall supra­molecular framework. Thus, the fluorine substituents do not constitute the primary packing motif but act as secondary acceptors that enhance the connectivity and stability of the crystal structure.

**Figure 3:**
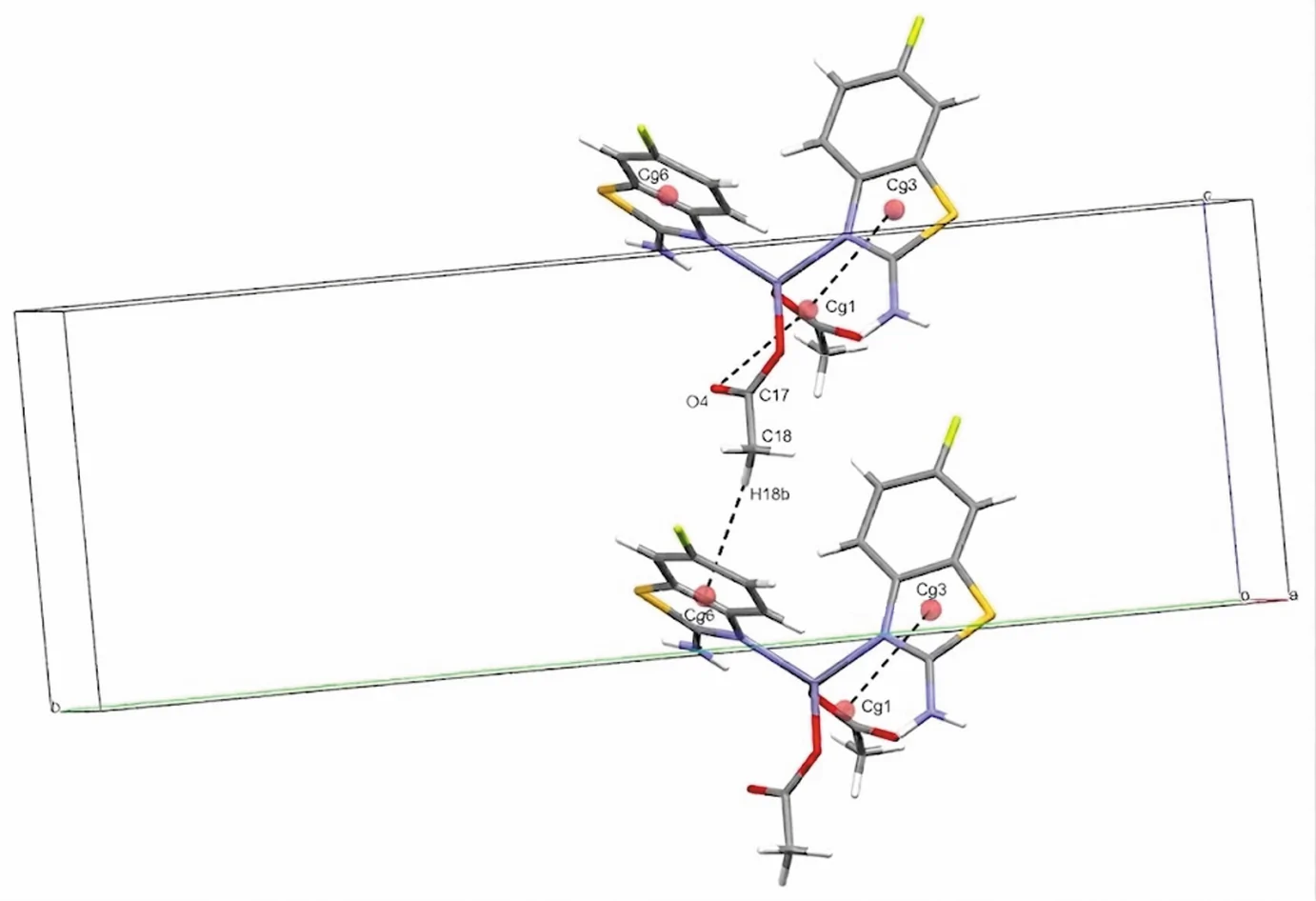
View of the π-related inter­molecular inter­actions in the crystal structure of the title compound, showing the offset π–π stacking inter­action (*Cg*1⋯*Cg*3), the C18—H18*B*⋯*Cg*6 C—H⋯π inter­action, and the C17—O4⋯*Cg*1 O⋯π contact. Selected inter­actions are shown as dashed lines.

The crystal packing is further reinforced by weak C—H⋯π and O⋯π inter­actions. The methyl group of the ac ligand forms a C18—H18*B*⋯*Cg*6 contact, where *Cg*6 is the centroid of the benzo­thia­zole ring system (S1/C1/N1/C2/C3/C4/C5/C6/C7). The corresponding geometric parameters are H18*B*⋯*Cg*6 = 2.972 (19) Å, C18⋯*Cg*6 = 3.565 (5) Å and C18—H18*B*⋯*Cg*6 = 121.1 (13)°, which are typical of weak but significant C—H⋯π inter­actions. In addition, the carbonyl oxygen atom O4 is involved in an O⋯π inter­action with the centroid *Cg*1 of the coordination plane (Zn1/O1/C15/O2), with an O4⋯*Cg*1 separation of 3.075 (4) Å (Fig. 3 ▸). Although individually weak, these contacts complement the hydrogen-bonding network and contribute to the efficient packing of the mol­ecules within the crystal.

A further characteristic feature of the crystal structure is the presence of a π–π inter­action between centroids *Cg*1 and *Cg*3, where *Cg*3 corresponds to the thia­zole ring (S2/C8/N3/C9/C14). The centroid-to-centroid distance of 3.7559 (19) Å falls within the range typically associated with offset π–π stacking inter­actions. This arrangement promotes favourable overlap of the π-electron systems while minimizing steric repulsion, thereby providing additional stabilization to the crystal packing.

## 4. Hirshfeld surface

**Figure 4:**
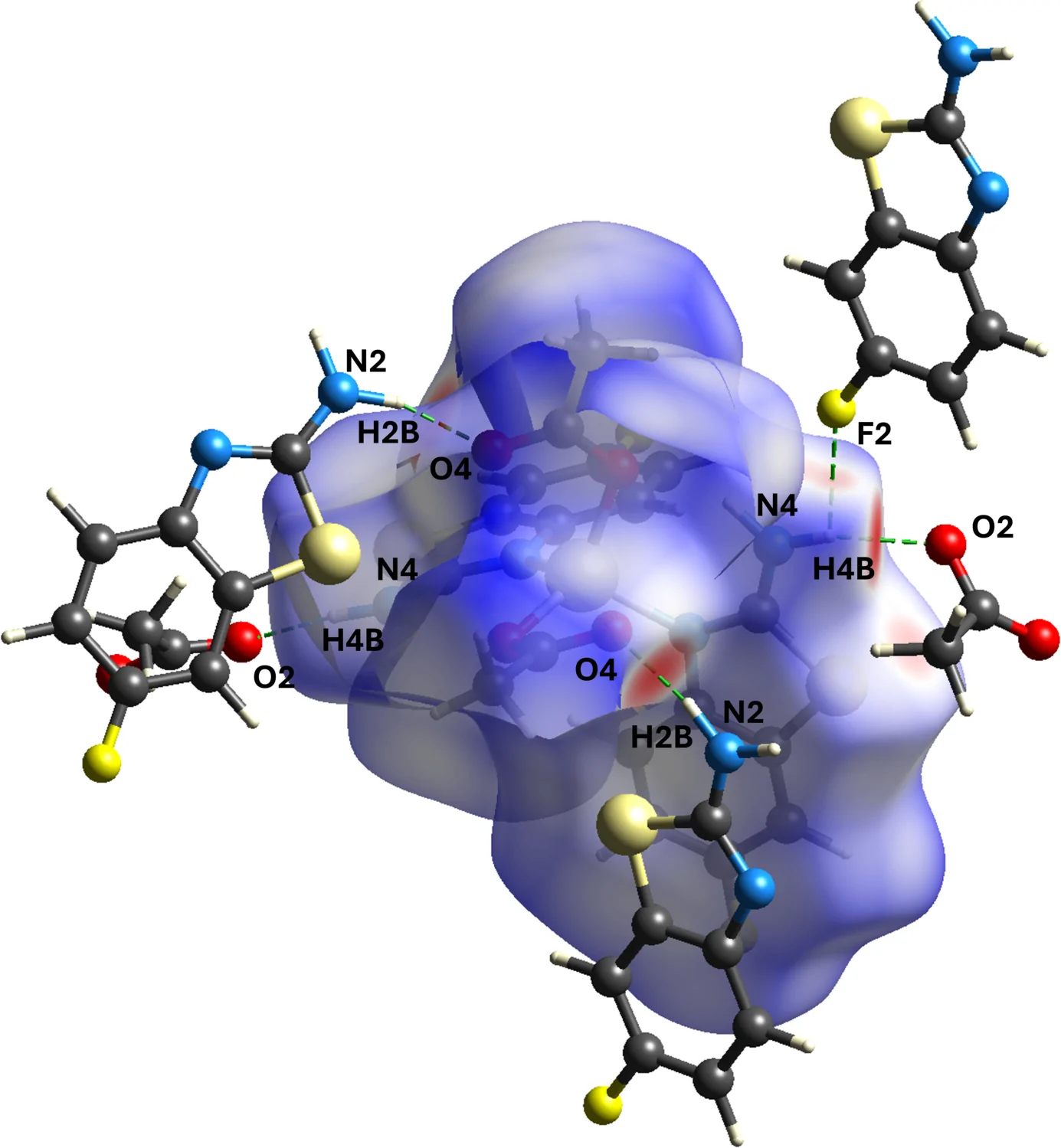
Hirshfeld surface of the title compound mapped over *d*_norm_, showing the inter­molecular contacts responsible for the crystal packing.

To obtain a deeper insight into the inter­molecular inter­actions governing the crystal packing, a Hirshfeld surface analysis was carried out using *CrystalExplorer21.5* (Spackman *et al.*, 2021 ▸). The Hirshfeld surface mapped over *d*_norm_ is shown in Fig. 4 ▸. The Hirshfeld surface has a total area of 444.97 Å^2^ and an enclosed volume of 517.52 Å^3^. The most intense red regions on the *d*_norm_ surface are localized around the non-coordinating oxygen atoms of the *ac* ligands and the amino groups of the benzo­thia­zole mol­ecules, corresponding to the shortest inter­molecular N—H⋯O hydrogen bonds identified in the crystal structure. Less pronounced red spots are observed around the fluorine atoms, confirming their involvement in weaker N—H⋯F inter­actions that complement the classical hydrogen-bonding network.

**Figure 5:**
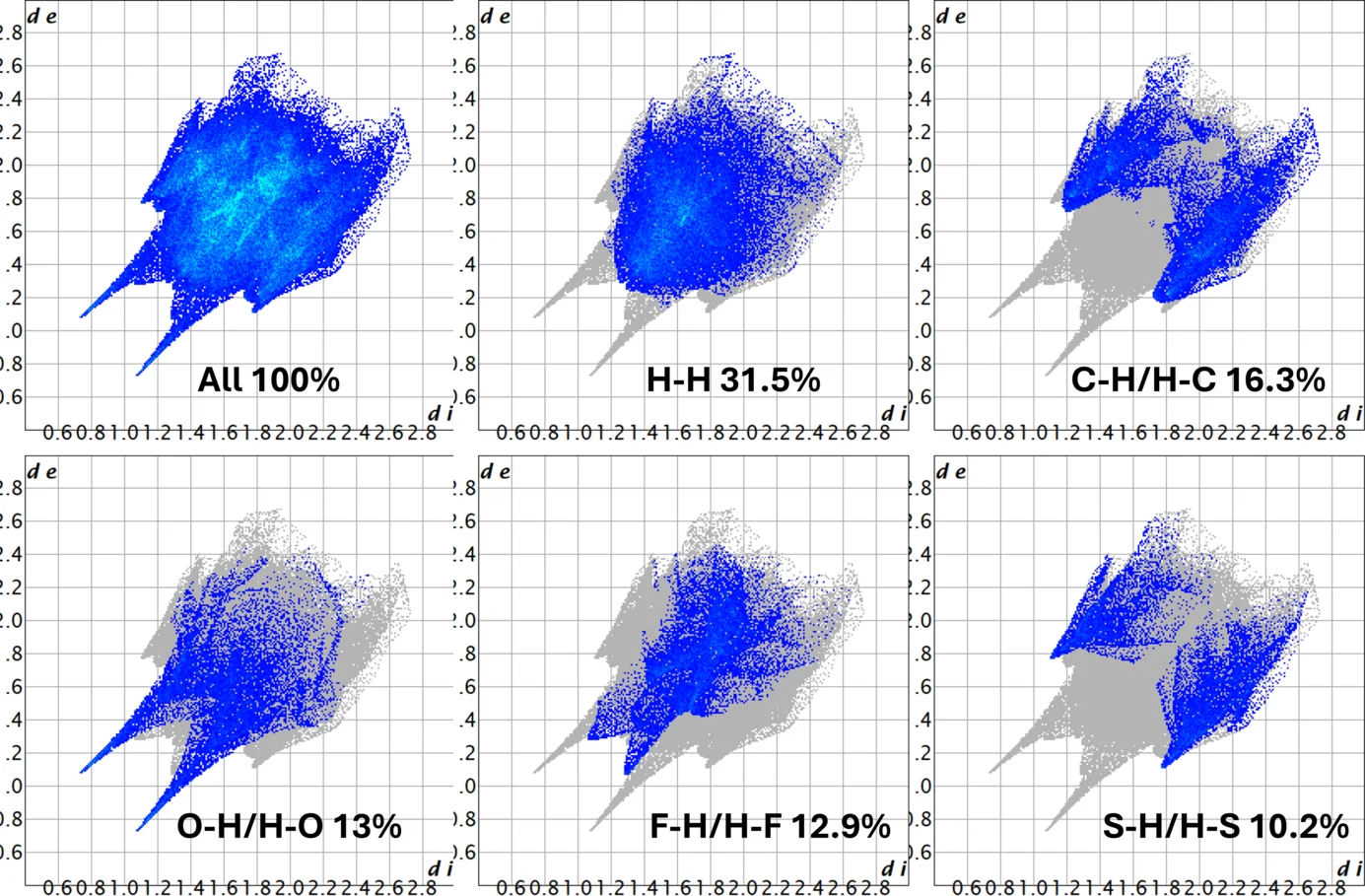
Two-dimensional fingerprint plots of the title compound showing the overall contacts and the individual contributions of H⋯H, C⋯H/H⋯C, O⋯H/H⋯O, F⋯H/H⋯F and S⋯H/H⋯S inter­actions to the Hirshfeld surface.

The two-dimensional fingerprint plots reveal that H⋯H contacts make the largest contribution to the Hirshfeld surface, accounting for 31.5% of the total surface area (Fig. 5 ▸). This predominance is characteristic of hydrogen-rich mol­ecular crystals and mainly reflects the close packing of neighbouring mol­ecules. The second-largest contribution arises from C⋯H/H⋯C contacts (16.3%), which are associated primarily with the C—H⋯π inter­actions between the acetate methyl groups and the aromatic benzo­thia­zole fragments. Although individually weak, these inter­actions contribute significantly to the stabilization of the crystal packing.

The O⋯H/H⋯O contacts contribute 13.0% to the Hirshfeld surface and are directly associated with the classical N—H⋯O hydrogen bonds forming the principal supra­molecular framework. Their characteristic sharp spikes on the fingerprint plots indicate the presence of strong directional hydrogen bonds. A comparable contribution is observed for F⋯H/H⋯F contacts (12.9%), demonstrating that the fluorine substituents participate actively in the inter­molecular inter­action network. Although fluorine is generally considered a weak hydrogen-bond acceptor, its involvement in the bifurcated N—H⋯(O,F) hydrogen bond highlights its important auxiliary role in reinforcing the hydrogen-bonded framework generated by the stronger N—H⋯O inter­actions.

The contribution of S⋯H/H⋯S contacts reaches 10.2%, reflecting numerous van der Waals contacts involving the sulfur atoms of the benzo­thia­zole rings. The remaining inter­actions make considerably smaller contributions, including S⋯C/C⋯S (3.5%), C⋯C (2.6%), C⋯F/F⋯C (2.5%), O⋯S/S⋯O (1.9%), F⋯N/N⋯F (1.5%), F⋯O/O⋯F (1.3%), N⋯H/H⋯N and F⋯S/S⋯F (each 1.0%), N⋯S/S⋯N (0.6%), and C⋯N/N⋯C, C⋯O/O⋯C, and F⋯F (each 0.1%). Although individually insignificant, these weak contacts collectively contribute to the overall stabilization of the crystal packing.

## 5. Database survey

A search of the Cambridge Structural Database (CSD, Version 2026.2.0; Groom *et al.*, 2016 ▸) revealed only three structurally related transition-metal complexes containing benzo­thia­zole-based ligands. Two entries, NOGZUG and NOHBAP (Sahoo *et al.*, 2014 ▸), are Cu^II^ complexes featuring cyclo­metallated imidazolidine–benzo­thia­zole ligands, while QAYKOU (Akhter *et al.*, 2022 ▸) is a Ru^II^ η^6^-*p*-cymene complex containing an amino­benzo­thia­zole derivative. All of these structures differ significantly from the title compound in both coordination mode and metal coordination geometry. To the best of our knowledge, no closely related Zn^II^ complex containing two neutral AFBT ligands together with two monodentately coordinated acetate anions has previously been reported.

## 6. Synthesis and crystallization

**Figure 6:**
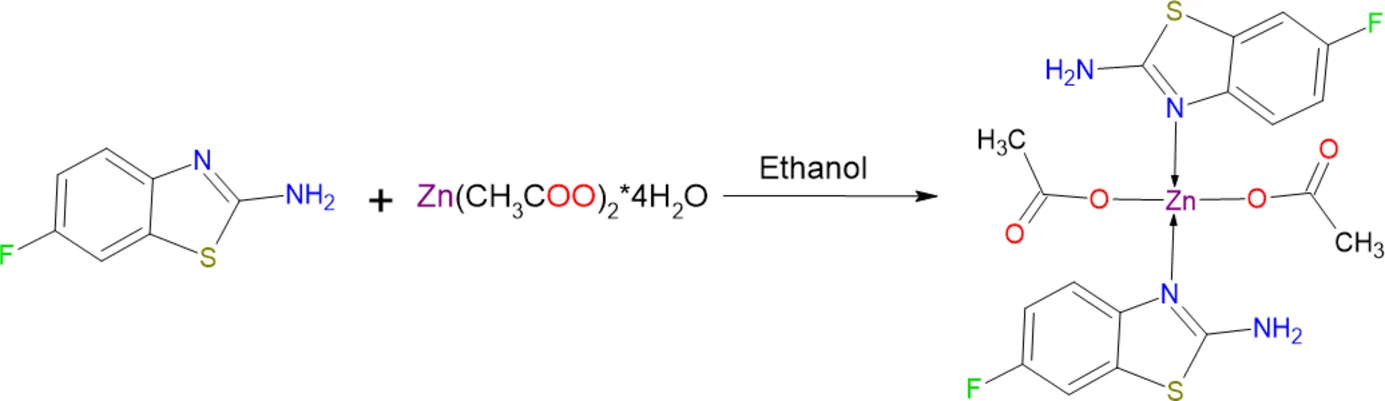
Reaction scheme.

The following solutions were prepared: (*a*) an ethano­lic solution of a Zn(CH_3_COO)_2_·4H_2_O (1.0 mmol) and (*b*) an ethano­lic solution of AFBT (2.0 mmol). Solution (*a*) was added to solution (*b*), and the resulting mixture was stirred at room tem­per­ature for 12 h using magnetic stirring. A crystalline precipitate was formed, filtered off, washed several times with ethanol and dried in air. Since the obtained material was readily soluble in di­methyl­formamide (DMF), it was recrystallized from this solvent. No poor solvent was used to induce crystallization; instead, the product precipitated directly from the ethano­lic reaction mixture because of its limited solubility in ethanol, yielding well-formed whitish single crystals suitable for X-ray diffraction analysis and further physicochemical investigations. The reaction scheme is shown in Fig. 6 ▸.

## 7. Refinement

Crystal data, data collection and structure refinement details are summarized in Table 3 ▸. All non-hydrogen atoms were refined anisotropically. Hydrogen atoms bonded to carbon atoms were positioned geometrically and refined using a riding model with C—H = 0.93–0.96 Å and *U*_iso_(H) = 1.2*U*_eq_(C) for aromatic carbon atoms and 1.5*U*_eq_(C) for methyl groups. Hydrogen atoms attached to nitro­gen atoms were located from difference-Fourier maps and refined with restrained N—H distances of 0.86 Å, with *U*_iso_(H) = 1.2*U*_eq_(N).

## Supplementary Material

Crystal structure: contains datablock(s) I. DOI: 10.1107/S2056989026009163/jp2034sup1.cif

Structure factors: contains datablock(s) I. DOI: 10.1107/S2056989026009163/jp2034Isup2.hkl

CCDC reference: 2584886

Additional supporting information:  crystallographic information; 3D view; checkCIF report

## Figures and Tables

**Table 1:** Selected geometric parameters (Å, °)

Zn1—O1	1.977 (3)	O1—C15	1.272 (5)
Zn1—O3	1.954 (2)	O3—C17	1.270 (4)
Zn1—N1	2.038 (3)	O4—C17	1.224 (5)
Zn1—N3	2.045 (3)	O2—C15	1.262 (5)
			
O3—Zn1—O1	119.78 (12)	N3—Zn1—O1	112.87 (11)
N1—Zn1—O1	104.15 (12)	N3—Zn1—O3	102.93 (10)
N1—Zn1—O3	109.13 (11)	N3—Zn1—N1	107.53 (11)

**Table 2:** Hydrogen-bond geometry (Å, °) *Cg*1 and *Cg*6 are the centroids of the Zn1/O1/C15/O2 and S1/N1/C1–C7 rings, respectively.

*D*—H⋯*A*	*D*—H	H⋯*A*	*D*⋯*A*	*D*—H⋯*A*
N2—H2B⋯O4^i^	0.86 (1)	1.97 (1)	2.806 (5)	162 (1)
N4—H4B⋯F2^ii^	0.86 (1)	2.44 (1)	2.910 (6)	115 (1)
N4—H4B⋯O2^iii^	0.86 (1)	2.07 (1)	2.890 (4)	160 (1)
C18—H18B⋯*Cg*6^iv^	0.96 (2)	2.97 (2)	3.565 (5)	121 (1)
C17—O4⋯*Cg*1	1.22 (1)	3.08 (1)	3.234 (4)	86 (1)

Symmetry codes: (i) 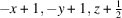; (ii) 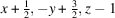; (iii) 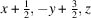; (iv) 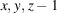.

**Table 3:** Experimental details

Crystal data	
Chemical formula	[Zn(C_2_H_3_O_2_)_2_(C_7_H_5_FN_2_S)_2_]
*M*_r_	519.88
Crystal system, space group	Orthorhombic, *Pna*2_1_
Temperature (K)	273
*a*, *b*, *c* (Å)	8.26379 (13), 27.6342 (3), 9.21400 (12)
*V* (Å^3^)	2104.14 (5)
*Z*	4
Radiation type	Cu *K*α
μ (mm^−1^)	3.95
Crystal size (mm)	0.37 × 0.28 × 0.25

Data collection	
Diffractometer	XtaLAB Synergy, Single source at home/near, HyPix3000
Absorption correction	Multi-scan (*CrysAlis PRO*; Rigaku OD, 2023 ▸)
*T*_min_, *T*_max_	0.336, 1.000
No. of measured, independent and observed [*I* ≥ 2σ(*I*)] reflections	19982, 4036, 3829
*R*_int_	0.063
(sin θ/λ)_max_ (Å^−1^)	0.616

Refinement	
*R*[*F*^2^ > 2σ(*F*^2^)], *wR*(*F*^2^), *S*	0.040, 0.113, 1.03
No. of reflections	4036
No. of parameters	282
No. of restraints	1
H-atom treatment	H-atom parameters constrained
Δρ_max_, Δρ_min_ (e Å^−3^)	0.38, −0.60
Absolute structure	Hooft et al. (2010 ▸)
Absolute structure parameter	0.002 (12)

Computer programs: *CrysAlis PRO* (Rigaku OD, 2023 ▸), *SHELXT* (Sheldrick, 2015 ▸), *OLEX2.refine* (Bourhis *et al.*, 2015 ▸) and *OLEX2* (Dolomanov *et al.*, 2009 ▸).

## supplementary crystallographic information

### Diacetatobis(2-amino-6-fluoro-1,3-benzothiazole-κN3)zinc(II) Crystal data

**Table:**

[Zn(C_2_H_3_O_2_)_2_(C_7_H_5_FN_2_S)_2_]	*D*_x_ = 1.641 Mg m^−^^3^
*M**_r_* = 519.88	Cu *K*α radiation, λ = 1.54184 Å
Orthorhombic, *P**n**a*2_1_	Cell parameters from 4970 reflections
*a* = 8.26379 (13) Å	θ = 3.7–71.1°
*b* = 27.6342 (3) Å	µ = 3.95 mm^−^^1^
*c* = 9.21400 (12) Å	*T* = 273 K
*V* = 2104.14 (5) Å^3^	Rhombohedral, clear whiteish colourless
*Z* = 4	0.37 × 0.28 × 0.25 mm
*F*(000) = 1055.915	

### Diacetatobis(2-amino-6-fluoro-1,3-benzothiazole-κN3)zinc(II) Data collection

**Table:**

XtaLAB Synergy, Single source at home/near, HyPix3000 diffractometer	3829 reflections with *I*≥ 2σ(*I*)
ω scans	*R*_int_ = 0.063
Absorption correction: multi-scan (CrysAlisPro; Rigaku OD, 2023)	θ_max_ = 71.7°, θ_min_ = 3.2°
*T*_min_ = 0.336, *T*_max_ = 1.000	*h* = −10→10
19982 measured reflections	*k* = −33→26
4036 independent reflections	*l* = −11→11

### Diacetatobis(2-amino-6-fluoro-1,3-benzothiazole-κN3)zinc(II) Refinement

**Table:**

Refinement on *F*^2^	Primary atom site location: dual
Least-squares matrix: full	H-atom parameters constrained
*R*[*F*^2^ > 2σ(*F*^2^)] = 0.040	*w* = 1/[σ^2^(*F*_o_^2^) + (0.0835*P*)^2^] where *P* = (*F*_o_^2^ + 2*F*_c_^2^)/3
*wR*(*F*^2^) = 0.113	(Δ/σ)_max_ = 0.001
*S* = 1.03	Δρ_max_ = 0.38 e Å^−^^3^
4036 reflections	Δρ_min_ = −0.60 e Å^−^^3^
282 parameters	Absolute structure: Hooft et al. (2010)
1 restraint	Absolute structure parameter: 0.002 (12)
26 constraints	

### Diacetatobis(2-amino-6-fluoro-1,3-benzothiazole-κN3)zinc(II) Special details

**Table:**

Refinement. The structure was solved by direct methods and refined by full-matrix least-squares on F^2^.

### Diacetatobis(2-amino-6-fluoro-1,3-benzothiazole-κN3)zinc(II) Fractional atomic coordinates and isotropic or equivalent isotropic displacement parameters (Å^2^)

**Table:**

	*x*	*y*	*z*	*U*_iso_*/*U*_eq_	
Zn1	0.63340 (5)	0.623334 (12)	0.39621 (5)	0.03898 (13)	
S2	0.72351 (14)	0.77996 (3)	0.52443 (10)	0.0590 (3)	
S1	0.72502 (15)	0.48713 (3)	0.66695 (11)	0.0644 (3)	
O1	0.3990 (3)	0.61134 (9)	0.3732 (3)	0.0533 (6)	
O3	0.7725 (3)	0.62617 (8)	0.2249 (3)	0.0451 (5)	
N1	0.7101 (4)	0.56872 (9)	0.5279 (3)	0.0432 (6)	
N3	0.6824 (3)	0.68720 (9)	0.4992 (3)	0.0392 (5)	
F1	1.2953 (4)	0.54886 (14)	0.8032 (4)	0.0932 (9)	
F2	0.4525 (8)	0.75495 (15)	1.0179 (4)	0.1439 (19)	
O4	0.6401 (4)	0.56292 (12)	0.1497 (5)	0.0902 (13)	
O2	0.4259 (4)	0.67805 (10)	0.2456 (3)	0.0654 (7)	
N2	0.4771 (4)	0.52090 (11)	0.5138 (4)	0.0626 (9)	
H2a	0.4271 (4)	0.54166 (11)	0.4607 (4)	0.0751 (11)*	
H2b	0.4299 (4)	0.49437 (11)	0.5382 (4)	0.0751 (11)*	
N4	0.8032 (4)	0.72560 (10)	0.2979 (4)	0.0555 (8)	
H4a	0.8114 (4)	0.69899 (10)	0.2500 (4)	0.0667 (10)*	
H4b	0.8368 (4)	0.75225 (10)	0.2603 (4)	0.0667 (10)*	
C17	0.7393 (4)	0.59503 (13)	0.1281 (4)	0.0479 (8)	
C8	0.7392 (4)	0.72552 (10)	0.4282 (4)	0.0419 (7)	
C9	0.6236 (4)	0.69968 (13)	0.6361 (4)	0.0452 (7)	
C2	0.8639 (4)	0.56674 (12)	0.5905 (4)	0.0472 (8)	
C16	0.1759 (6)	0.6390 (2)	0.2183 (7)	0.0813 (16)	
H16a	0.114 (2)	0.6150 (14)	0.269 (4)	0.122 (2)*	
H16b	0.123 (3)	0.6698 (6)	0.226 (5)	0.122 (2)*	
H16c	0.1849 (6)	0.6301 (19)	0.1179 (16)	0.122 (2)*	
C15	0.3419 (4)	0.64213 (12)	0.2842 (4)	0.0474 (7)	
C5	1.1534 (5)	0.5536 (2)	0.7335 (5)	0.0662 (11)	
C14	0.6342 (5)	0.74928 (14)	0.6671 (5)	0.0546 (9)	
C4	1.1284 (5)	0.5957 (2)	0.6536 (6)	0.0689 (12)	
H4	1.2085 (5)	0.6192 (2)	0.6478 (6)	0.0827 (14)*	
C3	0.9818 (5)	0.60183 (15)	0.5827 (5)	0.0595 (10)	
H3	0.9630 (5)	0.62987 (15)	0.5295 (5)	0.0714 (11)*	
C7	0.8947 (5)	0.52462 (13)	0.6702 (4)	0.0544 (9)	
C11	0.5019 (7)	0.6876 (2)	0.8684 (5)	0.0823 (15)	
H11	0.4590 (7)	0.6673 (2)	0.9391 (5)	0.0987 (18)*	
C10	0.5564 (5)	0.66893 (14)	0.7380 (5)	0.0588 (9)	
H10	0.5480 (5)	0.63597 (14)	0.7190 (5)	0.0705 (11)*	
C1	0.6246 (4)	0.52965 (12)	0.5575 (4)	0.0463 (8)	
C12	0.5122 (8)	0.7365 (2)	0.8919 (6)	0.0893 (16)	
C6	1.0398 (6)	0.51766 (16)	0.7425 (5)	0.0659 (11)	
H6	1.0594 (6)	0.48958 (16)	0.7953 (5)	0.0791 (14)*	
C13	0.5779 (8)	0.76845 (16)	0.7965 (5)	0.0783 (14)	
H13	0.5848 (8)	0.80134 (16)	0.8171 (5)	0.0940 (17)*	
C18	0.8288 (7)	0.5981 (3)	−0.0116 (5)	0.0884 (17)	
H18a	0.925 (3)	0.5785 (14)	−0.0062 (19)	0.133 (2)*	
H18b	0.761 (2)	0.5866 (17)	−0.0889 (9)	0.133 (2)*	
H18c	0.859 (5)	0.6311 (4)	−0.030 (3)	0.133 (2)*	

### Diacetatobis(2-amino-6-fluoro-1,3-benzothiazole-κN3)zinc(II) Atomic displacement parameters (Å^2^)

**Table:**

	*U*^11^	*U*^22^	*U*^33^	*U*^12^	*U*^13^	*U*^23^
Zn1	0.0520 (2)	0.02471 (19)	0.0402 (2)	−0.00300 (13)	0.0056 (2)	0.00357 (17)
S2	0.0945 (7)	0.0323 (4)	0.0502 (5)	−0.0112 (4)	0.0182 (5)	−0.0093 (3)
S1	0.0973 (7)	0.0342 (4)	0.0616 (5)	0.0019 (4)	0.0018 (6)	0.0195 (4)
O1	0.0536 (12)	0.0375 (11)	0.0689 (19)	0.0022 (9)	0.0021 (13)	0.0025 (12)
O3	0.0573 (13)	0.0375 (12)	0.0404 (12)	−0.0056 (9)	0.0056 (10)	−0.0060 (9)
N1	0.0580 (16)	0.0279 (12)	0.0437 (14)	0.0009 (11)	0.0044 (12)	0.0064 (11)
N3	0.0520 (14)	0.0313 (11)	0.0343 (12)	−0.0046 (10)	0.0064 (11)	0.0016 (10)
F1	0.0766 (17)	0.121 (3)	0.0819 (19)	0.0238 (17)	−0.0143 (16)	0.020 (2)
F2	0.257 (5)	0.100 (3)	0.075 (2)	0.020 (3)	0.092 (3)	−0.0093 (19)
O4	0.104 (3)	0.0625 (19)	0.104 (3)	−0.0374 (18)	0.022 (2)	−0.043 (2)
O2	0.0765 (18)	0.0472 (14)	0.0725 (19)	0.0039 (13)	0.0058 (17)	0.0103 (13)
N2	0.078 (2)	0.0347 (13)	0.075 (2)	−0.0137 (14)	−0.0018 (19)	0.0155 (15)
N4	0.091 (2)	0.0297 (12)	0.0464 (16)	−0.0151 (14)	0.0216 (17)	−0.0044 (12)
C17	0.0562 (19)	0.0389 (16)	0.0485 (18)	0.0035 (13)	0.0039 (15)	−0.0134 (14)
C8	0.0596 (18)	0.0242 (13)	0.0418 (18)	−0.0048 (11)	0.0059 (13)	−0.0026 (11)
C9	0.059 (2)	0.0412 (16)	0.0354 (16)	0.0011 (13)	0.0044 (14)	−0.0006 (13)
C2	0.068 (2)	0.0344 (16)	0.0388 (17)	0.0100 (13)	0.0079 (14)	0.0065 (13)
C16	0.056 (2)	0.081 (3)	0.108 (4)	0.006 (2)	−0.011 (3)	−0.039 (3)
C15	0.0567 (18)	0.0388 (17)	0.0466 (18)	0.0095 (14)	0.0070 (15)	−0.0027 (15)
C5	0.064 (2)	0.084 (3)	0.050 (2)	0.018 (2)	−0.0005 (19)	0.012 (2)
C14	0.074 (3)	0.0451 (19)	0.044 (2)	−0.0015 (16)	0.0128 (17)	−0.0036 (16)
C4	0.059 (2)	0.079 (3)	0.069 (3)	−0.0004 (19)	0.005 (2)	0.015 (3)
C3	0.063 (2)	0.0509 (19)	0.065 (2)	0.0026 (16)	0.0052 (18)	0.0197 (18)
C7	0.084 (2)	0.0404 (17)	0.0386 (18)	0.0153 (16)	0.0086 (17)	0.0085 (15)
C11	0.118 (4)	0.078 (3)	0.051 (3)	0.007 (3)	0.032 (3)	0.018 (2)
C10	0.076 (3)	0.0508 (19)	0.0498 (19)	0.0034 (18)	0.0124 (19)	0.0103 (16)
C1	0.068 (2)	0.0300 (15)	0.0408 (17)	−0.0015 (13)	0.0076 (15)	0.0037 (13)
C12	0.136 (5)	0.081 (3)	0.051 (2)	0.017 (3)	0.029 (3)	−0.011 (3)
C6	0.086 (3)	0.062 (2)	0.049 (2)	0.027 (2)	0.005 (2)	0.0136 (18)
C13	0.132 (4)	0.054 (2)	0.049 (2)	0.008 (3)	0.026 (3)	−0.0112 (19)
C18	0.101 (4)	0.116 (5)	0.048 (2)	0.002 (4)	0.013 (3)	−0.025 (3)

### Diacetatobis(2-amino-6-fluoro-1,3-benzothiazole-κN3)zinc(II) Geometric parameters (Å, º)

**Table:**

Zn1—O1	1.977 (3)	C9—C14	1.403 (5)
Zn1—O3	1.954 (2)	C9—C10	1.383 (5)
Zn1—N1	2.038 (3)	C2—C3	1.377 (5)
Zn1—N3	2.045 (3)	C2—C7	1.399 (5)
S2—C8	1.751 (3)	C16—H16a	0.9600
S2—C14	1.729 (4)	C16—H16b	0.9600
S1—C7	1.744 (4)	C16—H16c	0.9600
S1—C1	1.757 (4)	C16—C15	1.503 (6)
O1—C15	1.272 (5)	C5—C4	1.392 (7)
O3—C17	1.270 (4)	C5—C6	1.369 (7)
N1—C2	1.397 (5)	C14—C13	1.386 (6)
N1—C1	1.319 (4)	C4—H4	0.9300
N3—C8	1.330 (4)	C4—C3	1.387 (6)
N3—C9	1.395 (4)	C3—H3	0.9300
F1—C5	1.343 (5)	C7—C6	1.386 (6)
F2—C12	1.361 (6)	C11—H11	0.9300
O4—C17	1.224 (5)	C11—C10	1.383 (6)
O2—C15	1.262 (5)	C11—C12	1.371 (8)
N2—H2a	0.8600	C10—H10	0.9300
N2—H2b	0.8600	C12—C13	1.358 (8)
N2—C1	1.306 (5)	C6—H6	0.9300
N4—H4a	0.8600	C13—H13	0.9300
N4—H4b	0.8600	C18—H18a	0.9600
N4—C8	1.312 (5)	C18—H18b	0.9600
C17—C18	1.487 (6)	C18—H18c	0.9600
			
Cg1···Cg3	3.7559 (19)		
			
O3—Zn1—O1	119.78 (12)	C16—C15—O1	124.1 (4)
N1—Zn1—O1	104.15 (12)	C16—C15—O2	115.7 (4)
N1—Zn1—O3	109.13 (11)	C4—C5—F1	117.6 (5)
N3—Zn1—O1	112.87 (11)	C6—C5—F1	119.9 (4)
N3—Zn1—O3	102.93 (10)	C6—C5—C4	122.4 (4)
N3—Zn1—N1	107.53 (11)	C9—C14—S2	110.5 (3)
C14—S2—C8	89.73 (17)	C13—C14—S2	127.6 (3)
C1—S1—C7	89.56 (17)	C13—C14—C9	121.9 (4)
C15—O1—Zn1	108.7 (2)	H4—C4—C5	120.6 (3)
C17—O3—Zn1	114.4 (2)	C3—C4—C5	118.8 (4)
C2—N1—Zn1	123.9 (2)	C3—C4—H4	120.6 (3)
C1—N1—Zn1	124.3 (2)	C4—C3—C2	120.5 (4)
C1—N1—C2	111.7 (3)	H3—C3—C2	119.8 (2)
C8—N3—Zn1	121.9 (2)	H3—C3—C4	119.8 (3)
C9—N3—Zn1	124.3 (2)	C2—C7—S1	109.8 (3)
C9—N3—C8	111.8 (3)	C6—C7—S1	128.4 (3)
H2b—N2—H2a	120.0	C6—C7—C2	121.7 (4)
C1—N2—H2a	120.0	C10—C11—H11	120.5 (3)
C1—N2—H2b	120.0	C12—C11—H11	120.5 (3)
H4b—N4—H4a	120.0	C12—C11—C10	119.0 (4)
C8—N4—H4a	120.0	C11—C10—C9	119.4 (4)
C8—N4—H4b	120.0	H10—C10—C9	120.3 (2)
O4—C17—O3	121.5 (4)	H10—C10—C11	120.3 (3)
C18—C17—O3	117.5 (4)	N1—C1—S1	114.4 (3)
C18—C17—O4	121.0 (4)	N2—C1—S1	119.6 (3)
N3—C8—S2	114.1 (2)	N2—C1—N1	126.0 (3)
N4—C8—S2	119.5 (2)	C11—C12—F2	118.8 (5)
N4—C8—N3	126.4 (3)	C13—C12—F2	117.0 (5)
C14—C9—N3	113.8 (3)	C13—C12—C11	124.3 (4)
C10—C9—N3	127.0 (3)	C7—C6—C5	117.6 (4)
C10—C9—C14	119.2 (3)	H6—C6—C5	121.2 (2)
C3—C2—N1	126.5 (3)	H6—C6—C7	121.2 (2)
C7—C2—N1	114.5 (3)	C12—C13—C14	116.3 (4)
C7—C2—C3	119.0 (4)	H13—C13—C14	121.9 (3)
H16b—C16—H16a	109.5	H13—C13—C12	121.9 (3)
H16c—C16—H16a	109.5	H18a—C18—C17	109.5
H16c—C16—H16b	109.5	H18b—C18—C17	109.5
C15—C16—H16a	109.5	H18b—C18—H18a	109.5
C15—C16—H16b	109.5	H18c—C18—C17	109.5
C15—C16—H16c	109.5	H18c—C18—H18a	109.5
O2—C15—O1	120.2 (4)	H18c—C18—H18b	109.5
			
Zn1—O1—C15—O2	−11.6 (3)	F1—C5—C6—C7	179.2 (4)
Zn1—O1—C15—C16	168.2 (3)	F2—C12—C11—C10	177.4 (6)
Zn1—O3—C17—O4	−7.8 (3)	F2—C12—C13—C14	−178.3 (6)
Zn1—O3—C17—C18	174.0 (3)	N2—C1—S1—C7	179.5 (3)
Zn1—N1—C2—C3	4.9 (4)	N2—C1—N1—C2	−179.7 (4)
Zn1—N1—C2—C7	−176.3 (3)	N4—C8—S2—C14	179.6 (4)
Zn1—N1—C1—S1	176.9 (2)	N4—C8—N3—C9	179.6 (4)
Zn1—N1—C1—N2	−3.4 (3)	C8—S2—C14—C9	1.1 (2)
Zn1—N3—C8—S2	164.4 (2)	C8—S2—C14—C13	−178.8 (4)
Zn1—N3—C8—N4	−15.8 (3)	C8—N3—C9—C14	1.1 (3)
Zn1—N3—C9—C14	−163.0 (3)	C8—N3—C9—C10	−180.0 (3)
Zn1—N3—C9—C10	15.9 (4)	C9—C14—C13—C12	0.1 (5)
S2—C8—N3—C9	−0.2 (3)	C9—C10—C11—C12	1.6 (6)
S2—C14—C9—N3	−1.5 (3)	C2—C3—C4—C5	−0.5 (5)
S2—C14—C9—C10	179.5 (3)	C2—C7—S1—C1	0.7 (3)
S2—C14—C13—C12	180.0 (6)	C2—C7—C6—C5	−0.0 (4)
S1—C7—C2—N1	−0.5 (3)	C14—C9—C10—C11	−0.3 (5)
S1—C7—C2—C3	178.4 (3)	C14—C13—C12—C11	1.3 (8)
S1—C7—C6—C5	−177.2 (4)	C4—C5—C6—C7	−1.0 (6)
S1—C1—N1—C2	0.7 (3)	C4—C3—C2—C7	−0.5 (5)
N1—C2—C3—C4	178.3 (4)	C3—C2—N1—C1	−178.9 (4)
N1—C2—C7—C6	−178.1 (3)	C3—C2—C7—C6	0.7 (4)
N1—C1—S1—C7	−0.8 (3)	C3—C4—C5—C6	1.2 (6)
N3—C8—S2—C14	−0.5 (3)	C7—C2—N1—C1	−0.1 (4)
N3—C9—C14—C13	178.4 (4)	C10—C9—C14—C13	−0.6 (5)
N3—C9—C10—C11	−179.2 (5)	C10—C11—C12—C13	−2.3 (7)
F1—C5—C4—C3	−178.9 (4)	C1—S1—C7—C6	178.1 (3)

### Diacetatobis(2-amino-6-fluoro-1,3-benzothiazole-κN3)zinc(II) Hydrogen-bond geometry (Å, º)

*Cg*1 and *Cg*6 are the centroids of the Zn1/O1/C15/O2 and S1/N1/C1–C7 rings, respectively.

**Table:**

*D*—H···*A*	*D*—H	H···*A*	*D*···*A*	*D*—H···*A*
N2—H2B···O4^i^	0.86 (1)	1.97 (1)	2.806 (5)	162 (1)
N4—H4B···F2^ii^	0.86 (1)	2.44 (1)	2.910 (6)	115 (1)
N4—H4B···O2^iii^	0.86 (1)	2.07 (1)	2.890 (4)	160 (1)
C18—H18B···Cg6^iv^	0.96 (2)	2.97 (2)	3.565 (5)	121 (1)
C17—O4···Cg1	1.22 (1)	3.08 (1)	3.234 (4)	86 (1)

Symmetry codes: (i) −*x*+1, −*y*+1, *z*+1/2; (ii) *x*+1/2, −*y*+3/2, *z*−1; (iii) *x*+1/2, −*y*+3/2, *z*; (iv) *x*, *y*, *z*−1.
